# Supplementary material for: Majorana bound states from exceptional points in non-topological superconductors
Source: Sci Rep. 2016 Feb 11;6:21427. doi: 10.1038/srep21427 (PMC4750004; doi:10.1038/srep21427)
Supplement: Supplementary Information [file srep21427-s1.pdf]

Supplementary Information for 'Majorana bound states from exceptional points in non-topological superconductors'

Pablo San-Jose[1], Jorge Cayao[1], Elsa Prada[2], and Ramon Aguado[1,*]

[1]Instituto de Ciencia de Materiales de Madrid (ICMM), Consejo Superior de Investigaciones Científicas (CSIC), Cantoblanco, 28049 Madrid, Spain
[2]Departamento de Física de la Materia Condensada, Instituto de Ciencia de Materiales Nicolás Cabrera (INC), and Condensed Matter Physics Center (IFIMAC), Universidad Autónoma de Madrid, Cantoblanco, 28049 Madrid, Spain

ABSTRACT

In this supplemental material we discuss the robustness of the exceptional-point Majorana bound states presented in the main text against finite length and interaction effects.

1 Methods

Transport across the NS junction is computed using the nanowire model for a Rashba wire,

$$\begin{aligned} H_{\mathrm{S}} &= (2t-\mu_S)\sum_{\sigma n} c^\dagger_{\sigma n}c_{\sigma n} + \sum_{\sigma n}\Delta c^\dagger_{\sigma n}c^\dagger_{\bar\sigma n} + \mathrm{H.c} \\ &\quad - \sum_{\sigma,\langle n,n'\rangle} t\, c^\dagger_{\sigma n'}c_{\sigma n} - i\sum_{\sigma,\sigma'\langle n,n'\rangle} t^{\mathrm{SO}}_{n'-n} c^\dagger_{\sigma' n'}\sigma^y_{\sigma'\sigma}c_{\sigma n} \\ &\quad + \sum_{\sigma,\sigma' n} B\, c^\dagger_{\sigma' n}\sigma^x_{\sigma'\sigma}c_{\sigma n} \end{aligned} \tag{1}$$

with the non-proximized normal section (N) modelled by the same Hamiltonian, albeit with $\Delta = 0$ and a μ_N in place of μ_S. The normal contact transmission $T_N^{(n)}$ of each incoming mode is computed by also setting $\Delta = 0$ on the proximised (S) side, and using the standard Green's function scheme. One first splits the system into a left lead (with μ_N), a right lead (with μ_S), and a central section (the interface with a non-uniform profile $\mu(x)$ that transitions from μ_N into μ_S) coupled to the leads through operators $V_{N/S}$. The total conductance $\mathscr{G}$ of the M incoming modes is then given by Caroli's formula[1]

$$\mathscr{G} = \sum_n^M T_N^{(n)} = 4\mathscr{G}_0 \mathrm{Tr}\left[\Gamma_N G \Gamma_S G^\dagger\right] \tag{2}$$

where $\mathscr{G}_0 = e^2/h$, G is the dressed retarded Green's function of the central region, $\Gamma_{N/S} = (\Sigma_{N/S} + \Sigma^\dagger_{N/S})/2$ is the decay operator into the left/right leads, $\Sigma_{N/S} = V^\dagger_{N/S} g_{N/S} V_{N/S}$ is the corresponding self energies, and $g_{N/S}$ is the surface Green's function of the decoupled leads.

The poles of the scattering matrix presented in the main text are given, close to the origin of the complex plane, by the eigenvalues of non-Hermitian Hamiltonian $H_S + \Sigma(\omega = 0)$, where H_S is the (Hermitian) Hamiltonian of a sufficiently long segment of the wire containing the junction, and Σ is the self-energy from the remaining wire (the reservoir), that is computed numerically as described above.

The average normal transmission per mode is defined as $T_N = \mathscr{G}/(M\mathscr{G}_0)$. The values given in the main text were computed for Zeeman $B = 0$. T_N depends on the detailed spatial interpolation profile $\mu(x)$ across the interface. An abrupt interface has a smaller transmission than a smooth one, due to the mismatch in Fermi velocity between the two sides. In a real sample, the smoothness of such depletion profile is controlled by geometric parameters of the gating used to deplete the normal side (typically the superconducting side will be difficult to deplete due to screening by the parent superconductor). T_N can also be controlled in a real device by adding a pinch-off gate close to the contact. This possibility is modelled by suppressing a single hopping term t precisely at the contact, where $\Delta(x)$ abruptly jumps from zero to Δ. The combination of mismatch and pinch-off allows to sweep T_N from zero to one.

2 Finite wire effects

The results presented in the main text for the NS junction in a proximised Rashba wire assumed ideal, infinitely long N and the S sides of the wire. In this section we discuss the corrections that should be expected in real samples with finite lengths L_S and L_N, see 1a. We show that the emergence of EP-MBSs only weakly depends on these corrections, and is dominated by the properties of the NS contact itself, as described in the main text.

While a finite S length L_S of the proximised wire has no influence in the spectrum of the sample as long as it exceeds its coherence length, the same is not obviously true for L_N, particularly if the contact resistance between the N portion of the wire and the macroscopic normal reservoir is large. In such case, the N side will behave like a 1D quantum box between the reservoir and the S side (both modelled with the same $\mu_S \gg \Delta$), rather than as an infinite 1D reservoir for the S side. However, this has little impact on the stabilisation of the EP-MBSs for realistic parameters. In Fig. 1b we show the differential conductance dI/dV from a tunnel probe, for the same case as in Fig. 4f in the main text, albeit with a realistic $L_N = 1.5\mu$m. The total normal transmission is reduced to $T_N = 0.5$ by increasing the reservoir-N contact resistance (the NS junction is still assumed transparent). While considerable structure then arises in the dI/dV due to Fabry-Perot interference effects at finite bias voltage V, the sharp EP-MBS peak (red) is only weakly affected, and remains pinned to zero energy. Plotting the dI/dV versus the reservoir-N transmission, Fig. 1c, we see that the EP-MBSs actually remains sharp for any value of T_N, unlike the conventional Andreev bound states at finite V. This pattern is replicated also for the differential conductance from the reservoir (analogous to Fig. 4h in the main text), shown in Fig. 1(d,e). While the finite L_N produces considerable structure at finite bias, the sharp dip at zero bias is insensitive to the value of L_N and T_N, as long as the NS interface remains close to transparency.

3 Interaction effects

We now briefly discuss our expectations concerning the robustness of our conclusions against interactions in the normal side. Using renormalization group arguments, Fidkowski et al have demonstrated in Ref.[2] that the universal low-energy properties of NS junctions (with N described as a Luttinger liquid) are governed by fixed points of either perfect normal reflection or perfect Andreev reflection. In the case of junctions with a trivial superconductor, like the ones discussed here, they demonstrate that perfect Andreev reflection is unstable for strong negative interactions ($g < 2$ in the Luttinger liquid picture). In this case, the low-energy properties of the junction are governed by perfect normal reflection (trivial behaviour) which results in $dI/dV|_{V\to 0} = 0$, as expected. The finite voltage conductance vanishes at small voltages as the power law

$$G \sim (V/V^*)^{2(2/g-1)},$$

with V^* a crossover voltage that defines the renormalization group flow from perfect Andreev reflection to perfect normal reflection. This conductance reduction results in a sharp dip in the dI/dV, as measured from the helical wire. Note that this dip already occurs without interactions, see Fig. 4h. As we have discussed in the main text, such dip ultimately arises from the formation of a localized dark state, measurable as a quantized dI/dV peak from a third probe, and whose residual lifetime is related to the amplitude of Andreev reflections. This dip has been connected to the so-called Beri degeneracy that predicts a $dI/dV = 0$ from a single mode reservoir at $V \to 0$ if the topology is trivial,[2–6] and has been shown to appear even in topologically non-trivial wires of finite length (see Fig. 4g in the main text). The role of interactions, therefore, is to add corrections to that residual lifetime. For an infinitely long helical wire and a perfectly transparent contact, interactions will give a lower bound on the residual decay rate of the dark state.

For a finite-length wire, however, as is relevant for realistic junctions, the Luttinger liquid corrections to the dI/dV are only valid for voltages above $\tilde{V} \sim \hbar v_F/L_N$, the reason being that at low enough energies all physical quantities should be more sensitive to the long distance part of the lead (which is a noninteracting Fermi liquid reservoir with $g = 1$). Thus, one expects the conductance to cross over to the noninteracting value for voltages $V \lesssim \tilde{V}$. This sets a range of voltages where we expect our results to be robust even in the presence of negative interactions in the helical wire. For the parameters of Fig. 1(d), $\mu_N = 0.14meV$ and $L_N = 1.5\mu m$, we estimate $\tilde{V} = \hbar v_F/L_N \sim 25\mu eV = 0.1\Delta$ which is much larger than the small residual lifetime around $V \sim 0$. This sets a realistic voltage range $V \lesssim \tilde{V}$ where the renormalization group flow towards normal reflection is cut off. In this voltage range, we expect that our results of EP-MBS in highly transparent trivial junctions should be robust even in the presence of strong negative interactions.

Positive interactions, on the other hand, make the perfect Andreev reflection fixed point stable. Such fixed point stabilised by strong attractions offers an alternative scenario, similar to the one discussed here, where Majorana zero modes may exist in junctions with trivial superconductors.[2]

References

1. Caroli, C., Combescot, R., Nozières, P. & Saint-James, D. Direct calculation of the tunneling current. *J. Phys. C: Solid St. Phys* **4**, 916–929 (1971).

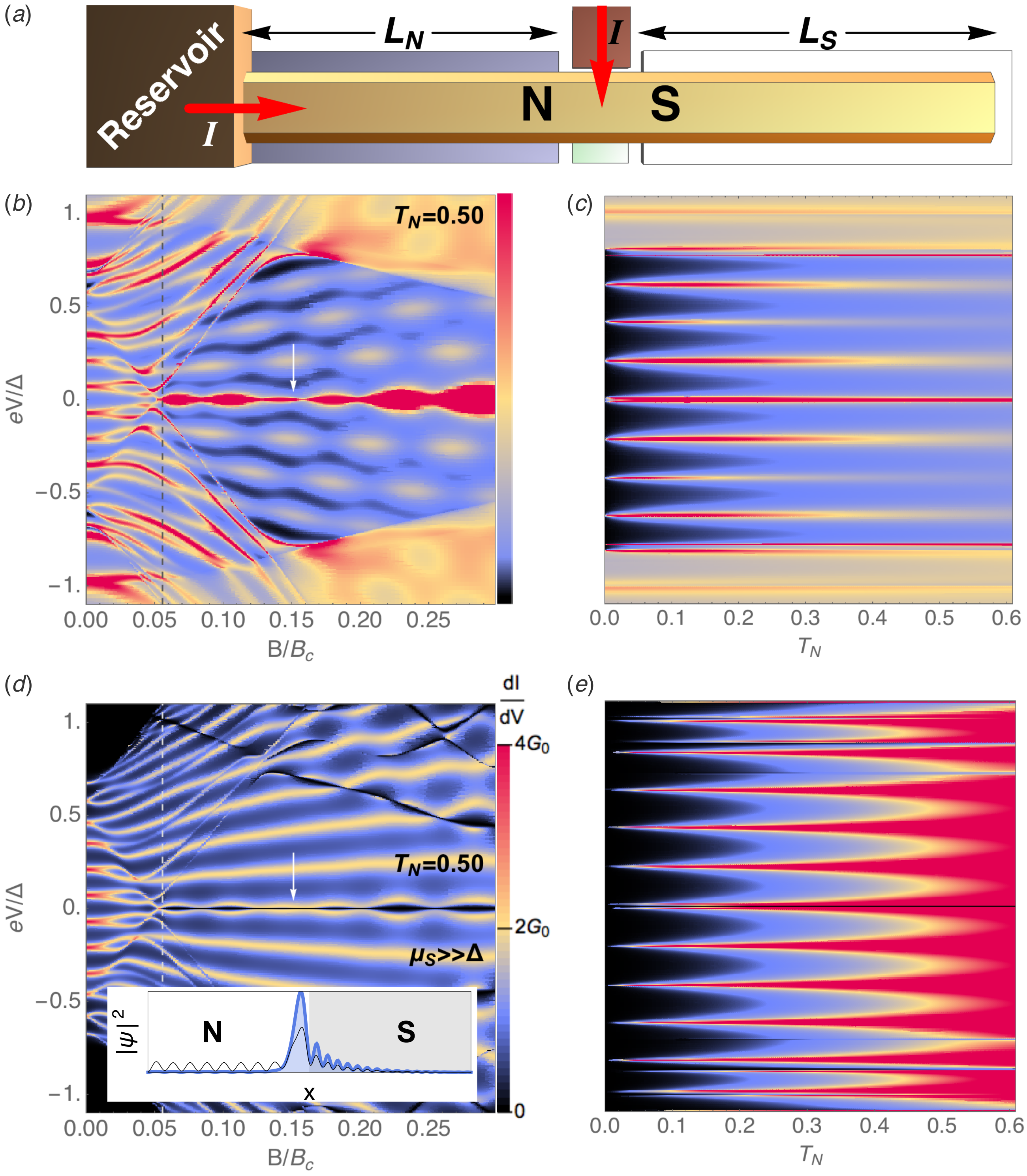

Figure 1. (a) A sketch of a realistic sample with finite wire lengths, L_N and L_S. The overall transmission of the junction is a combination of the reservoir-N resistance and the NS resistance. (b) The dI/dV conductance from a third tunneling probe for parameters like in Fig. 4f of the main text, albeit with $L_N = 1.5\mu$m and a reduced transmission $T_N = 0.5$ coming from the reservoir-N contact resistance. (c) The same dI/dV at a fixed B (white arrow in panel b), versus T_N. Unlike the finite bias Andreev resonances, the zero-bias peak from the EP-MBSs is largely insensitive to T_N and L_N. (d,e) Differential conductance from the metallic reservoir, instead of the probe (compare to Fig. 4h in the main text). In the inset to (d), spatial profile of the lowest eigenmode of the wire (at the arrow), for $T_N = 0.5$ (thick blue line) and $T_N = 0$ (thin black line), showing localization of the state at the junction. For the parameters of the simulation, the decay rate of the zero-energy scattering state for $T_N = 0.5$ corresponds to $\sim 0.1\mu eV$.

2. Fidkowski, L., Alicea, J., Lindner, N. H., Lutchyn, R. M. & Fisher, M. P. A. Universal transport signatures of majorana fermions in superconductor-luttinger liquid junctions. *Phys. Rev. B* **85**, 245121 (2012).

3. Béri, B. Dephasing-enabled triplet andreev conductance. *Phys. Rev. B* **79**, 245315 (2009).

4. Pikulin, D. I., Dahlhaus, J. P., Wimmer, M., Schomerus, H. & Beenakker, C. W. J. A zero-voltage conductance peak from weak antilocalization in a majorana nanowire. *New Journal of Physics* **14**, 125011 (2012).

5. Pikulin, D. I. & Nazarov, Y. V. Two types of topological transitions in finite majorana wires. *Phys. Rev. B* **87**, 235421 (2013).

6. Ioselevich, P. A. & Feigel'man, M. V. Tunneling conductance due to a discrete spectrum of andreev states. *New Journal of Physics* **15**, 055011 (2013).
